# Decreased sexual desire and distress symptoms: an analysis among urban Chinese women

**DOI:** 10.1093/sexmed/qfag018

**Published:** 2026-04-19

**Authors:** Lan Luo, Tong Fu, Jingjing Huang, Huafang Li

**Affiliations:** Shanghai Mental Health Center, Shanghai Jiao Tong University School of Medicine, Shanghai 200030, China; Shanghai Mental Health Center, Shanghai Jiao Tong University School of Medicine, Shanghai 200030, China; Shanghai Mental Health Center, Shanghai Jiao Tong University School of Medicine, Shanghai 200030, China; Shanghai Mental Health Center, Shanghai Jiao Tong University School of Medicine, Shanghai 200030, China; Shanghai Key Laboratory of Psychotic Disorders, Shanghai 200030, China

**Keywords:** hypoactive sexual desire disorder, sexual desire, sexual distress, women

## Abstract

**Background:**

Despite China’s national women’s development policies aimed at enhancing female sexual health, research on hypoactive sexual desire disorder (HSDD) remains insufficient, hindering its clinical diagnosis and treatment.

**Aim:**

We aimed to explore the primary self-reported contributing factors for decreased sexual desire among Chinese women of childbearing age and to characterize their experiences of sexual distress symptoms.

**Methods:**

An online cross-sectional survey was conducted from April to July 2020. A total of 3443 valid responses (valid response rate 82.6%) from adult Chinese women were analyzed.

**Outcomes:**

Data included demographics and the Decreased Sexual Desire Screener (DSDS) adapted to assess HSDD symptoms.

**Results:**

Respondents were primarily Chinese urban women of childbearing age (median 26, interquartile range 23-30). “Stress and fatigue” was the primary self-reported contributing factor to decreased sexual desire (53.6%, n = 1846). The prevalence of this factor remained high after standardization for demographic variables (standardized rates ranged from 50.1% to 55.2%). Furthermore, this factor was more prevalent among women with higher levels of education (compared to “middle school,” adjusted ORs ranged from 1.52 to 1.86 across education subgroups). Specifically, intense work pressure was associated with decreased sexual desire attributed to various factors (compared to “moderate,” adjusted ORs ranged from 1.29 to 1.86). Overall, 18.8% (n = 648) were not satisfied with their level of sexual desire or interest, 40.2% (n = 1383) were bothered by their decreased sexual desire, and 63.6% (n = 2190) wanted it to increase. A total of 5.7% (n = 195) of our participants were at high risk for HSDD (defined by our criteria).

**Clinical Implications:**

This study identified key factors influencing decreased sexual desire in Chinese urban women of childbearing age, challenges a simple deficit-based model of HSDD, and highlights a significant unmet need for sexual well-being services.

**Strengths and Limitations:**

This first large-scale study on the drivers and related distress symptoms of decreased sexual desire in Chinese women addresses a critical knowledge gap. Caution is warranted, however, in interpreting the findings as the cross-sectional design and online sample limit generalizability, and the assessment tools used have not been rigorously validated in a Chinese population.

**Conclusion:**

Decreased sexual desire was prevalent in Chinese urban women of childbearing age and was mostly associated with self-reported stress and fatigue, especially work pressure; notably, the majority of Chinese women desired higher level of sexual desire.

## Introduction

Female sexual health is integral to overall well-being^1^; yet remains a globally neglected field of medicine.^2^ Although national policies, such as the “Healthy China 2030” plan, prioritize women’s health to encourage childbirth, research and clinical services for female sexual dysfunction (FSD) in China remain inadequate. This deficiency contrasts with the well-established field of male sexual medicine, which offers numerous pharmacological treatments for conditions such as erectile dysfunction. This disparity is most evident in the case of hypoactive sexual desire dysfunction (HSDD), the most common FSD worldwide.^3^ HSDD is characterized by persistent low sexual desire and related clinically significant personal distress. It remains poorly understood and largely unaddressed in China, despite the existence of treatments in the United States and a few other countries.^4^^,^^5^ Current literature reveals two critical knowledge gaps: the primary biopsychosocial drivers of low desire among Chinese women are unclear,^6^ and sexual distress—the factor distinguishing a symptom from a clinical dysfunction—is routinely overlooked.^7^ Drawing on broader frameworks regarding cultural influence on reproductive mental health,^8^ it is possible that Chinese women exhibit distinct patterns in how sexual distress is experienced and expressed compared to Western cohorts. This underscores the need for localized empirical data.

### A significant knowledge gap regarding the etiology of low sexual desire in Chinese women hinders the diagnosis and treatment of HSDD

Although the high prevalence of low sexual desire among Chinese women is well-documented,^9^^,^^10^ its primary drivers and clinical characteristics remain unclear. Limited studies have linked sexual desire to age,^10^ education,^11^ gynecological surgeries,^12^ and metabolic status.^13^ However, existing evidence fails to reflect the full range or relative impact of these factors. This knowledge gap hinders clinicians from promptly identifying sexual desire dysfunctions and from screening for relevant etiologies. Furthermore, it complicates the development of effective sexual education and public science communication.

To address the multifaceted nature of decreased sexual desire and distress, this study utilizes the biopsychosocial model. This framework posits that female sexual function involves a complex interplay of biological predispositions, psychological factors (eg, stress, fatigue, mood), and social-contextual influences (eg, work pressure, relationship dynamics, educational background). This model provides a robust theoretical basis for investigating how these specific factors contribute to the prevalence of distressing low sexual desire among Chinese women and for understanding their sexual distress.

Building upon the biopsychosocial model, it is critical to identify the most salient factors affecting contemporary Chinese women. Conducted in 2020, this study captures the unique societal pressures of the COVID-19 pandemic, which likely amplified stressors related to work, family life, and general uncertainty. This context provides a pertinent setting to examine the influence of such pressures.

### Neglect of sexual distress symptoms obscures the clinical significance and heterogeneity of HSDD

Although low sexual desire is a prerequisite for HSDD, the dysfunction is defined by significant sexual distress. However, quantitative research in China has largely neglected this critical component. Existing studies addressing distress have found it to be common among Chinese women, but these investigations rely primarily on clinical or hospital-based samples^14^^,^^15^ and, therefore, fail to capture its prevalence and nature at the population level. Furthermore, the experience of distress among Chinese women may be culturally modulated. Qualitative studies in China suggest that some women do not perceive low desire as inherently problematic,^16^ which may be largely due to cultural narratives that value sexual modesty or restraint of desire.^17^ Thus, low desire alone may not necessarily result in personal distress for all Chinese women.

### Objectives and hypotheses

Therefore, the present study aims to address these gaps by investigating the following questions among Chinese women of childbearing age: (1) within a biopsychosocial framework, are psychosocial factors (eg, stress, fatigue) more prominent contributors to decreased sexual desire than biological or lifestyle factors during the COVID-19 pandemic? (2) is the prevalence of distressing decreased sexual desire lower than the rates observed in Western women (~10%^18^)? and (3) are specific demographic, lifestyle, and health factors significantly associated with decreased sexual desire?

## Materials and methods

### Sample and procedure

This cross-sectional study was approved by the Shanghai Mental Health Center-Institutional Review Board (Number: 2020-05) and adhered to the World Medical Association Declaration of Helsinki. To address potential stigma regarding sexual health, we employed a discreet online questionnaire distributed via WeChat, Weibo, and other online forums from April to July 2020. The survey began with an electronic informed consent form (ICF). Participants were informed of the study background, objectives, and their right to withdraw at any time; access to the questionnaire was granted only after they confirmed their voluntary participation. Inclusion required the capacity to comprehend and complete the survey. Quality control measures included monitoring response time and completion rates; data from male participants or minors were excluded.

To mitigate the selection bias inherent in online convenience sampling, we applied three strategies. First, we maximized sample diversity by disseminating the survey across varied platforms (including WeChat, Weibo, and various online forums) to reach distinct social circles. Second, to enhance representativeness, we performed post-stratification standardization against 2020 Chinese census data for key variables (age, education, marital status, and residence). Finally, we utilized multivariable logistic regression to control for confounders, thereby strengthening internal validity.

### Instruments

#### Demographic information questionnaire

Informed by the literature, we assessed factors associated with female sexual desire: (a) sociodemographics, including marital/cohabitation status, education, occupation,^19^ income, and residence (to enable post-stratification standardization); (b) lifestyle, specifically smoking^20^ and alcohol consumption^21^; and (c) health status, including self-reported gynecological diseases.^22^ Additionally, given the COVID-19 pandemic, we assessed major life events in the past year to account for significant acute stressors as potential confounders or predictors.

#### Questionnaire about symptoms of HSDD

HSDD symptoms were assessed using the Decreased Sexual Desire Screener (DSDS),^23^ a validated five-item, patient-reported instrument for diagnosing HSDD in pre- and postmenopausal women according to DSM-IV-TR and ISSWSH criteria.^24^ The first item of the original DSDS asks women whether they have previously experienced a satisfactory level of sexual desire to determine whether the condition is acquired or lifelong. We modified this item to focus on sexual satisfaction rather than the “acquired” vs. “lifelong” distinction. This adjustment draws upon the concept of sexual distress in the Female Sexual Distress Scale-Desire/Arousal/Orgasm (FSDS-DAO) to facilitate a more in-depth analysis of distress symptoms among Chinese women. The full questionnaire is provided below. Based on the original methodology,^23^ the high-risk population for HSDD was defined as participants who answered “no” to item 1 and “yes” to items 2 through 4. Decreased sexual desire accompanied by sexual distress in these women likely reaches a level requiring clinical attention.

**Table:** 

Questionnaire about Symptoms of Hypoactive Sexual Desire Disorder
(1) Are you satisfied with your level of sexual desire or interest recently?
(2) Has there been a decrease in your level of sexual desire or interest?
(3) Are you bothered by your decreased level of sexual desire or interest?
(4) Would you like your level of sexual desire or interest to increase?
(5) Please circle all the factors that you feel may be contributing to your current decrease in sexual desire or interest:
A. An operation, depression, injuries, or other medical condition
B. Medications, drugs, or alcohol you are currently taking
C. Pregnancy, recent childbirth, menopausal symptoms
D. Other sexual issues you may be having (pain, decreased arousal, or orgasm)
E. Your partner’s sexual problems
F. Dissatisfaction with your relationship or partner
G. Stress or fatigue

#### Sexual health attitude questionnaire

We also administered eight items assessing women’s sexual health attitude within this online study; further details are reported elsewhere.^25^

### Statistical analysis

Data analysis was performed from March to May 2022 using R version 4.1.3 (R Foundation for Statistical Computing, Vienna, Austria). We reported medians and quartiles for continuous variables and frequencies and percentages for categorical variables. Results were standardized by age, education, marital status (single, married, divorced, or widowed) and residence (eastern, central, western, and northeast; defined by the National Bureau of Statistics of China based on economic development) according to 2020 census data (https://www.stats.gov.cn/sj/pcsj/rkpc/7rp/indexch.htm).

Binary logistic regression was used to explore relationships between demographics and self-reported contributors to decreased sexual desire. In these models, contributors served as outcomes and demographics as predictors (selected via stepwise backward regression). We calculated adjusted odds ratios (aOR) with statistical significance set at *P* < 0.05. Age categories included “18-19” (n = 189; reference), “20-29” (n = 2247), “30-39” (n = 865), “40-49” (n = 109), and “50+” (n = 33). For *education*, “middle school” was the reference. With ~30 predictor variables, our sample size exceeds the recommended minimum of 1600.^26^

## Results

Of the 4168 responses collected (average time: 208 seconds), we excluded cases with response times <30 s (n = 13, 0.3%), age < 18 years (n = 61, 1.5%), missing answers to questions on sexual health attitudes and HSDD symptoms (n = 13, 0.3%), and male (n = 568, 13.6%) or unknown gender (n = 70, 1.7%). This yielded 3443 (82.6%) valid responses. Except for cohabitants, all variables contained missing data; rates were highest (3.3%-7.1%) for *age, education, physical* or *gynecological diseases*, and contributors to decreased desire. Remaining items had <0.5% missing data, excluding *number of children* (1.5%). We imputed missing values using logistic and polytomous regression.

Participant demographics (Table 1, Supplemental Table 1) indicate a predominantly young (median [interquartile range]: 26 [23-30]) and married (51.6%, n = 1776) sample. Most (86.1%, n = 2964) grew up in urban areas, with residence spanning all Chinese provincial administrative region except Tibet and Taiwan. Compared to 2020 census data, participants were younger and more educated; thus, the results primarily represent urban Chinese women of reproductive age. Overall, 195 (5.7%) women met the criteria for high HSDD risk.

**Table 1:** Demographics of participants.

Variables	N(%)/M(Q1-Q3)	Variables	N(%)/M(Q1-Q3)	Variables	N(%)/M(Q1-Q3)
**Age**	26 (23-30)	**Ethnic group**		Missing	4 (0.1%)
Missing	204 (5.9%)	Han^a^	3268 (94.9%)	**Current occupation**	
**Marital status**		Others	171 (5.0%)	Farmer	84 (2.4%)
Unmarried	1482 (43.0%)	Missing	4 (0.1%)	Worker	206 (6.0%)
Married	1776 (51.6%)	**Years of education**		Office staff	1243 (36.1%)
Widowed	66 (1.9%)	median (Q1-Q3)	15 (12-16)	Civil servant	391 (11.4%)
Divorced	82 (2.4%)	Missing	120 (3.5%)	Professional	627 (18.2%)
Separated	32 (0.9%)	**Education**		Self-employed	182 (5.3%)
Missing	5 (0.1%)	Out of school	27 (0.8%)	Retired	39 (1.1%)
**Duration of stable relationship**		Primary school	50 (1.5%)	Student	589 (17.1%)
≥12 months	2078 (60.4%)	Middle school	230 (6.7%)	Unemployed	37 (1.1%)
6-12 months	688 (20.0%)	High school	551 (16.0%)	Other	43 (1.2%)
1-6 months	627 (18.2%)	University	2122 (61.6%)	Missing	2 (0.1%)
Missing	50 (1.5%)	Postgraduate	459 (13.3%)	**Monthly household income per capita**	
**Number of children**		Missing	4 (0.1%)	≥¥5000	1881 (54.6%)
0	1673 (48.6%)	**Growth environment^b^**		¥3000-¥5000	1158 (33.6%)
1	1122 (32.6%)	Tier 1 city	743 (21.6%)	¥1000-¥3000	330 (9.6%)
2	523 (15.2%)	Tier 2 city	1228 (35.7%)	<¥1000	66 (1.9%)
>2	119 (3.5%)	Tier 3 and below cities	993 (28.8%)	Missing	8 (0.2%)
Missing	6 (0.2%)	Rural areas	475 (13.8%)		

^a^Han refers to the majority ethnic group in China, constituting over 90% of the population.

^b^The classification of city was based on Ranking of cities’ business attractiveness 2020.^27^^,^^28^

### Self-reported contributing factors of decreased sexual desire

Decreased sexual desire was prevalent, reported by 43.9% (n = 1510) of participants, with higher rates among women over 50 (66.7%, n = 22).

**Figure 1:**
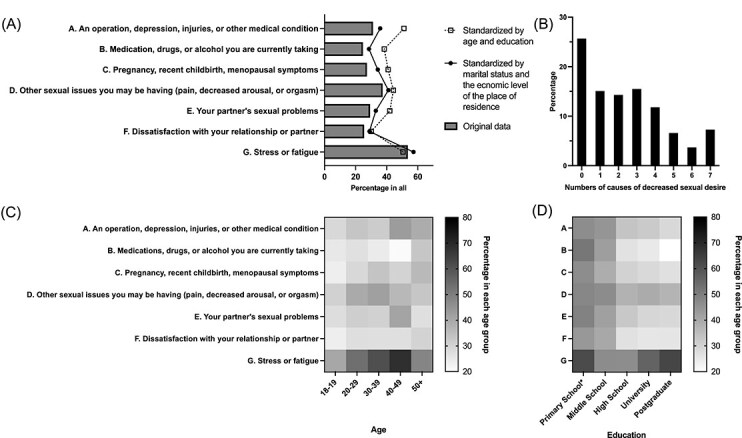
Self-reported contributing factors of decreased sexual desire in participants (N = 3443). ^*^This classification combines women with primary education and those who have dropped out of school.

Among self-reported contributory factors (Figure 1A), *stress or fatigue* (Factor G, 53.6%, n = 1846) was the most common, followed by *other sexual issues the women had* (Factor D, 37.4%, n = 1287). The majority of women (59.2%, n = 2039) reported multiple contributing factors (Figure 1B).

Standardization by marital status and residence yielded results similar to the unadjusted data, with primary factors persisting and proportions shifting by no ˃8%. In contrast, standardization by age and education revealed significant differences. The most common factors then became *an operation, depression, injuries, or other medical condition* (Factor A, 51.6%), *stress or fatigue* (Factor G, 50.2%), *the partner’s sexual problems* (Factor E, 44.3%), and *other sexual issues the women had* (Factor D, 44.1%).

Stratification by age (Figure 1C) and education (Figure 1D) highlighted specific trends. For women over 50, *medications, drugs, or alcohol* (Factor B, 36.4%, n = 12) and *menopausal symptoms* (Factor C, 42.4%, n = 14) were prominent. *Partners’ sexual issues* (Factor E) was prevalent among women in their forties (42.4%, n = 46). *Stress and fatigue* (Factor G) was strongly associated with decreased desire in middle-aged women (>50%), and remained common (>40%) in other age groups. Regarding education, lower attainment was associated with stronger links to most factors, except for *stress and fatigue* (Factor G), which remained the primary factor across nearly all education levels.

Binary logistic regression analyses (Table 2 and Supplementary Table 3A–G) identified demographic and lifestyle characteristics associated with the seven self-reported factors. The imputed distribution of predictor variables is presented in Supplementary Table 2. Significant correlates of *stress and fatigue* (Factor G) included university or above education background (compared to “middle school,” aORs ranged from 1.52 to 1.86), intense work pressure (compared to “moderate,” aOR = 1.86, 95%CI 1.59-2.19), major life events in the past year (compared to “no,” aOR = 1.65, 95%CI 1.20-2.28), drinking habits (compared to “never,” aORs ranged from 1.55 to 1.62), rural or third-tier/lower city backgrounds (compared to “tier 1 city”, aORs ranged from 1.34 to 1.35), and being married (compared to “unmarried,” aOR = 1.33, 95%CI 1.12-1.58). Conversely, women in tier 2 cities (compared to “tier 1 city,” aOR = 0.67, 95%CI 0.53-0.85) were less likely to report Factor G. Intense work pressure (compared to “moderate,” aORs ranged from 1.29 to 1.86) was also linked to various other factors, followed by illness, living with parents, and occasional smoking. Physical diseases (compared to “no”) were associated with organic factors (A, B, and C), whereas gynecological diseases (compared to “no”) or major life events (compared to “no”) correlated with psychological and relationship factors (D, E, F, and G). Partner-related factors (E and F) were more strongly associated with living with parents (compared to “no,” aORs ranged from 1.19 to 1.59) and living in non-nuclear families (compared to “nuclear family,” aORs ranged from 1.35 to 1.61). Additionally, civil servants, self-employed women, office staff, and professionals (compared to “students,” aORs ranged from 1.28 to 1.97) were more likely to report *other sexual issues* or *dissatisfaction with partners* (Factors D and F). Having children correlated with *pregnancy, childbirth, or menopausal symptoms* (Factor C). Finally, regarding medication (Factor B) attribution, higher education was negatively associated (compared to “middle school,” aORs ranged from 0.45 to 0.60), while civil servants, professionals (compared to “student,” aORs ranged from 1.48 to 1.59), smokers (compared to “never,” aORs ranged from 1.55 to 1.82), and women in non-first-tier cities (compared to “tier 1 city,” aORs ranged from 1.32 to 1.55) showed a positive association.

**Table 2:** Adjusted odds ratios (aOR) in logistic regression models for decreased sexual desire due to various contributing factors.

Variables	Levels	A	B	C	D	E	F	G
Ethic group (ref = Han)	Manchu	-	-	0.56^*^	-	-	-	-
Marriage (ref = Unmarried)	Divorced	-	-	2.82^***^	-	-	-	-
	Married	-	1.52^***^	1.53^***^	-	-	-	1.33^***^
	Widowed	-	-	2.03^*^	-	-	-	-
Education (ref = Middle school)	High School	-	0.58^**^	-	-	-	-	-
	Postgraduate	-	0.45^***^	-	-	-	-	1.86^***^
	University	-	0.60^**^	-	-	-	-	1.52^*^
Current occupation (ref = Student)	Civil servants	1.65^***^	1.48^*^	-	1.47^**^	-	1.89^***^	-
	Office staff	1.30	-	-	1.28^*^	-	1.36^*^	1.36^*^
	Self-employed	-	-	-	1.50^*^	-	1.59^*^	-
	Professionals	1.62^***^	1.59^**^	-	1.45^**^	-	1.97^***^	-
	Retired	-	-	-	-	-	2.99^*^	-
	Workers	2.09^***^	-	-	-	-	-	-
Trait (ref = Introverted)	Mixed	0.73^***^	0.73^**^	-	-	0.74^**^	0.71^***^	-
Smoking (ref = Never)	Every day	-	-	-	-	-	3.33^**^	-
	Occasionally	1.57^***^	1.82^***^	1.33^*^	1.44^***^	-	1.63^***^	-
	Often	-	1.55^*^	-	-	-	1.68^**^	0.49^***^
Drinking (ref = Never)	Occasionally	-	-	-	-	-	-	1.55^***^
	Often	1.64^*^	-	-	-	1.55^*^	-	1.62^*^
Monthly household income per capita (ref = ≥¥5 k)	￥3000-￥5000	-	-	-	1.18^*^	-	-	-
Dominant hand (ref = Right)	Left	-	-	1.29^*^	-	-	-	-
Work pressure (ref = Moderate)	Intense	1.30^***^	1.29^**^	-	1.40^***^	1.36^***^	1.37^***^	1.86^***^
Growth environment (ref = Tier 1 city)	Rural area	-	0.63^**^	-	-	-	-	1.35^*^
	Tier 3 and below cities	-	-	-	-	-	-	1.34^**^
Place of residence (ref = Tier 1 city)	New tier 1 city	-	1.39^*^	-	1.38^**^	-	-	-
	Other cities	-	-	0.54^*^	-	-	-	-
	Tier 2 city	-	1.32^*^	-	-	-	-	0.67^***^
	Tier 3 city	-	1.55^*^	-	1.37^*^	1.64^***^	-	-
	Tier 4 city	-	1.41^*^	-	1.34^*^	1.39^*^	-	-
	Tier 5 city	-	1.55^**^	-	-	1.63^**^	-	-
Family structure (ref = Nuclear family)	Reorganized family	-	1.83^***^	-	-	1.57^**^	1.61^**^	-
	Single-parent family	-	1.59^***^	-	-	1.35^*^	1.41^**^	-
Family relationships (ref = Harmonious)	Average	-	-	-	1.21^*^	1.31^**^	1.29^*^	-
Living with children (ref = No)	Yes	-	-	-	1.28^*^	1.22^*^	-	-
Living with parents (ref = No)	Yes	1.20^*^	1.36^***^	1.22^*^	-	1.19^*^	1.59^***^	-
Living with relatives (ref = No)	Yes	-	-	1.47^***^	-	-	-	-
Living with friends (ref = No)	Yes	-	1.38^*^	1.41^**^	-	-	1.50^**^	-
Living with others (ref = No)	Yes	2.73^**^	-	-	-	-	-	-
Number of children (ref = 0)	1	-	-	1.51^**^	-	-	-	-
	2	-	-	1.68^***^	-	-	-	-
	>2	-	-	1.90^**^	0.57^*^	-	-	-
Major events in the past year (ref = No)	Yes	-	-	-	1.54^**^	-	1.46^*^	1.65^**^
Physical disease (ref = No)	Yes	1.86^***^	1.36^*^	1.54^***^	-	-	-	-
Gynecological Disease (ref = No)	Yes	1.54^***^	-	-	2.30^***^	1.70^***^	1.60^***^	1.78^***^

^*^ *P* < .05.

^**^ *P* ≤ .01.

^***^ *P* ≤ .001.

### Sexual distress symptoms and expectation of sexual desire in Chinese women

#### Prevalence and characteristics of sexual distress symptoms

**Figure 2:**
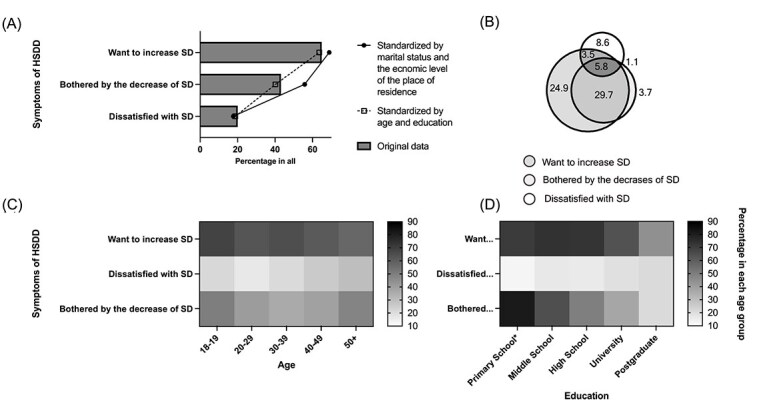
Sexual distress symptoms and expectation of sexual desire in participants (N = 3443). ^*^ This classification combines women with primary education and those who have dropped out of school. The Venn diagram (b) shows the percentage of women (N = 3443) reporting specific sexual distress symptoms.

A substantial portion of participants reported distress symptoms related to decreased sexual desire (Figure 2A). Specifically, 18.8% (n = 648) were dissatisfied with their sexual desire or interest, while 40.2% (n = 1383) were bothered by decreased desire. Based on our definition, 5.7% (n = 195) of the sample was identified as high-risk for HSDD. Notably, post-stratification adjustment for age and education significantly increased the standardized prevalence of women bothered by decreased desire, from 40.2% to 56.0% (Figure 2A).

#### The aspiration for increased desire as a noteworthy phenomenon

The discrepancy between reported distress symptoms and the aspiration for increased sexual desire was notable. While 40.2% of women were bothered by their decreased desire, a significantly larger proportion (63.6%, n = 2190) expressed a wish for increased sexual desire. Among those seeking increased desire, the vast majority (85.5%, n = 1873) were not dissatisfied with their sexual desire, and 39.0% (n = 854) reported neither current bother nor dissatisfaction (Figure 2B).

#### Sociodemographic correlates of sexual distress symptoms

Stratified analyses revealed distinct demographic patterns (Figure 2C and D). Bother regarding decreased desire was most prevalent among women with lower educational attainment, specifically those with primary schooling or less (81.8%, n = 63). Additionally, age followed a U-shaped distribution: women in the youngest (18-19) and oldest (>50) age groups were more likely to report bother than those in the middle age brackets.

## Discussion

This study represents one of the first large-scale investigations into factors contributing to decreased sexual desire and the characteristics of sexual distress symptoms among Chinese women of childbearing age. Our findings confirm that distressing decreased sexual desire is prevalent. Psychosocial factors—specifically stress and fatigue driven by work pressure—were the primary self-reported contributors to decreased sexual desire. The 5.7% prevalence of women at high risk for HSDD was slightly lower than rates reported in Western studies,^24^ but women generally wish to increase their sexual desire. Furthermore, the observed heterogeneous nature of sexual distress symptoms challenges deficit-based diagnostic models, highlighting the need for a culturally attuned, well-being-oriented approach to female sexual health in China.

### Self-reported contributing factors of decreased sexual desire

Consistent with the view of low sexual desire as multifactorial,^29^ most participants attributed their decreased desire to multiple factors (Figure 1B). The predominance of *stress and fatigue* (Figure 1A) supports our first hypothesis and aligns with prior qualitative findings.^30^ A robust association with intense work pressure (Table 2), particularly among highly educated women, reflects the work-life balance challenges in urban China.^31^ Significantly, family burden was not a major factor, suggesting that professional rather than domestic life is the primary stressor affecting this cohort’s sexual well-being. Given that decreased sexual desire can be a manifestation of depression—often triggered by stress—these patterns may serve as early indicators of depressive symptoms. Furthermore, our study refines the specific stressors linked to the decreased sexual desire in women during the pandemic compared to previous research.^32^

Relationship dissatisfaction (Factor F) was infrequently cited, whereas *other sexual issues* was common (Figure 1A). This pattern suggests participants may internalize sexual problems, attributing them to personal physiological or psychological deficits rather than relational dynamics (eg, perceiving lack of arousal as a personal failing rather than a result of insufficient stimulation). Although requiring further investigation, this underscores the need for cognitive evaluation and psychoeducation in clinical and research settings.

Stratified analysis and data standardization revealed important demographic nuances (Figure 1A). Partners’ sexual problems became more significant after age standardization, reflecting higher prevalence among women in their forties and fifties (Figure 1C), consistent with age-related increases in male sexual dysfunction.^33^ Similarly, women over 40 and those with lower education attainment were more likely to attribute decreased desire to medical conditions (Factor A), highlighting vulnerable groups requiring targeted healthcare interventions.

The data indicated the influence of the “family of origin”. Women from non-intact families of origin or those living with their parents were more likely to attribute decreased sexual desire to partner dissatisfaction (Table 2). This emphasizes the importance of addressing family dynamics in sex and couples therapy. Future research should explore the mechanisms linking parental and family structures to couples’ sexual relationships.

### Sexual distress symptoms and expectation of sexual desire in Chinese women

Our results suggest the high prevalence and complexity of sexual distress. Over 40% of participants reported being bothered by decreased sexual desire, underscoring the widespread psychological impact of these concerns. However, when using more stringent screening criteria, the prevalence of high-risk HSDD (5.7%) remains lower. This discrepancy suggests that while “bother” is a sensitive indicator of distress, the proportion of Chinese women experiencing decreased sexual desire with significant distress may be slightly lower than in Western countries.^18^ Notably, the percentage of women bothered by decreased sexual desire (40.2%) was more than double that of those dissatisfied with it (18.8%), with limited overlap. This indicates that distress is not monolithic; women may feel bothered (eg, confused or worried) without feeling dissatisfied, and vice versa. This aligns with findings that distress stems from various sources, such as confusion or perceived deviation from a norm, rather than dissatisfaction alone.^34^ Because alleviating distress is the primary goal of HSDD treatment, this heterogeneity warrants further exploration to refine clinical interventions.

Age and education were significantly linked to distress. Women over 50 and those aged 18-19 were more likely to be bothered by decreased sexual desire (Figure 2C), though likely for different reasons related to relationship duration and sexual attitudes. Conversely, higher education correlated with lower dissatisfaction or bother regarding sexual desire (Figure 2D). Educated women may derive satisfaction from non-sexual domains, offsetting desire-related concerns. These factors warrant further investigation to better understand sexual distress among Chinese women.

#### Beyond a deficit-based model: implications for clinical practice and sexual well-being

The discrepancy between women bothered by decreased desire (40.2%) and the larger group (63.6%) wishing for a higher level of desire has profound clinical implications. This suggests that the yearning for a higher level of sexual desire is a broader phenomenon than the endorsement of decreased desire or the presence of associated bother. For these women, the aspriration for a higher level of desire may represent a pursuit for passion, pleasure, and intimacy within their relationships rather than an effort to remediate a dysfunction. This finding challenges the deficit-based medical model, which defines problems solely by negative symptoms (ie, distress). Our results suggest a necessary paradigm shift toward a holistic model of sexual well-being that incorporates personal aspirations for sexual enhancement. This echoes the World Health Organization’s (WHO) position that sexual health should not be limited to addressing dysfunctions but should recognize the pursuit of satisfying, positive sexual experiences.^35^

These aspirations may reflect societal shifts in China regarding quality of life and personal fulfillment. Consequently, clinical inquiry must evolve; simply asking “Does your low desire bother you?” is insufficient. For example, a patient successfully treated for depression with selective serotonin reuptake inhibitors (SSRIs) who experiences reduced sexual desire might deny being “bothered” by reduced desire to avoid altering a medication vital for her mental stability, despite aspiring to a more fulfilling sexual life. The current HSDD diagnostic framework overlooks such needs.

A more empowering approach involves asking, “What are your personal goals for your sexual life?” This reframes the clinical encounter from fixing a dysfunction to collaborating toward optimal well-being. Such a shift is crucial for the nearly 40% of participants who desired a higher level of desire without formal distress—a silent majority whose needs currently remain unaddressed.

### Policy and public health implications

Our findings offer actionable implications for public health policy in China, particularly regarding the “Healthy China 2030” initiative and new population policies:

1. Integrate Female Sexual Well-being into National Health and Family Policies. The high prevalence of distressing decreased desire confirms it as a significant public health issue rather than a niche concern. Given the national focus on family harmony and birth rates, female sexual well-being should be recognized as pivotal to marital stability and quality of life. Policymakers must fund and integrate female sexual health services into national agendas, supported by destigmatization campaigns.

2. Mandate a Biopsychosocial Screening Model. Identifying work-related stress as the primary contributor demonstrates the inadequacy of purely biological approaches. Clinical guidelines for primary care, gynecology, and mental health services should require routine screening for psychosocial stressors—specially work pressure—in women with sexual complaints. First-line interventions should prioritize stress management and psychological counseling.

3. Develop Culturally Tailored Clinical Tools and Public Education. The observed heterogeneity of female sexual distress symptoms underscores the complexity of this issue among Chinese women, highlighting the need for localized research on the dysfunction’s natural history and the development and validation of relevant assessment tools. Public health education should shift from a focus on dysfunction to a positive message of sexual well-being.

4. Promote a Relational and Family-Centered Approach. The link between decreased desire and partner or family dynamics indicates that female sexual health cannot be treated in isolation. Policies should consider subsidizing couples counseling as a core component of sexual health services. This aligns with national goals of fostering healthy family relationships and a supportive environment for child-rearing.

### Limitations and future directions

While this study provides significant insights, its findings should be interpreted within the context of several limitations that suggest clear avenues for future research.

1. Sampling and Generalizability: Reliance on an online convenience sample introduces selection bias, potentially over-representing urban, digitally literate women willing to discuss sexuality. Although post-stratification standardization partially mitigated this issue, future research utilizing probability-based national sampling is necessary to validate these prevalence estimates. Additionally, as data were collected during the COVID-19 pandemic, the finding that “stress and fatigue” significantly impact female sexual desire requires validation in a non-pandemic context.
2. Study Design and Causality: The cross-sectional design identifies correlates but precludes causal inference. Furthermore, self-report data may be subject to recall or social desirability biases. Future longitudinal studies tracking the trajectory of sexual desire are needed to provide stronger evidence for causality and a deeper understanding of the dynamic nature of these factors.
3. Depth of Correlates: This preliminary exploration excluded key psychological and relational variables. Future research should incorporate validated measures of relational satisfaction, partner communication, sexual self-image, and attachment patterns to facilitate a comprehensive biopsychosocial model of decreased sexual desire within the Chinese context.
4. Measurement: Measurement choices shaped the scope of our findings. The questionnaires were adapted but have not yet been fully validated for Chinese women, and our survey did not comprehensively cover symptoms related to sexual distress. Because we did not assess symptom duration, our data offer indirect evidence for hypotheses regarding HSDD prevalence rather than definitive diagnostic rates. The DSDS follows DSM-IV-TR criteria, which align more closely with ICD-11 standards than with the DSM-5, in which desire and arousal dysfunctions are merged into Female Sexual Interest/Arousal Disorder (FSIAD). Consequently, our results are comparable to studies investigating sexual desire and distress symptoms, but not to those strictly utilizing DSM-5 criteria. Despite these limitations, we selected the DSDS because its conciseness facilitated a larger sample size and its alignment with the ICD-11 reflects current understanding of this dysfunction and avoids the controversy regarding the DSM-5’s FSIAD criteria.^36^
5. Understanding Sexual Distress and Education: Finally, while we noted the heterogeneity of sexual distress symptoms, we did not detail its causes or severity. The association between higher educational attainment and specific HSDD symptoms warrants further investigation. Future qualitative or mixed-methods studies should explore the mechanisms through which education influences women’s sexual health and agency to inform the design of equitable, effective sexuality education programs.

## Conclusions

In conclusion, decreased sexual desire is prevalent among Chinese women of childbearing age and is predominantly associated with self-reported stress and fatigue, particularly work pressure. Sexual distress presents as a heterogeneous symptom, and the majority of women express an aspiration for enhanced sexual well-being. These findings underscore the critical necessity of increasing clinical awareness and providing timely, culturally tailored interventions for this population.

## Supplementary Material

qfag018_supinfo(2)

## Data Availability

No. We collect sensitive information from participants.

## Code availability

Software application or custom code: No.
