# Supplementary material for: Decreased sexual desire and distress symptoms: an analysis among urban Chinese women
Source: Sex Med. 2026 Apr 19;14(3):qfag018. doi: 10.1093/sexmed/qfag018 (PMC13092269; doi:10.1093/sexmed/qfag018)
Supplement: qfag018_supinfo(2) [file qfag018_supinfo(2).docx]

**SUPPLEMENTAL INFORMATION**

**Supplemental Table 1** Demographic characteristics of participants (imputed)

|  |  |  | (Continued) |  |
| --- | --- | --- | --- | --- |
| Variables | Total (N=3443) |  | Variables | Total (N=3443) |
| **Age** |  |  | **Current occupation** |  |
| 18-19 | 189 (5.5%) |  | Students | 590 (17.1%) |
| 20-29 | 2247 (65.3%) |  | Farmers | 84 (2.4%) |
| 30-39 | 865 (25.1%) |  | Workers | 206 (6.0%) |
| 40-49 | 109 (3.2%) |  | Office staff | 1243 (36.1%) |
| ≥50 | 33 (1.0%) |  | Civil servants | 392 (11.4%) |
| **Education** |  |  | Professionals | 627 (18.2%) |
| Middle school | 230 (6.7%) |  | Self-employed | 182 (5.3%) |
| Out of school | 27 (0.8%) |  | Retired from illness | 18 (0.5%) |
| Primary school | 50 (1.5%) |  | Retired | 21 (0.6%) |
| High school | 552 (16.0%) |  | Unemployed | 37 (1.1%) |
| University | 2124 (61.7%) |  | Others | 43 (1.2%) |
| Postgraduate | 460 (13.4%) |  | **Smoking** |  |
| **Ethnic group** |  |  | Never | 2854 (82.9%) |
| Han | 3272 (95.0%) |  | Occasionally | 425 (12.3%) |
| Manchu | 102 (3.0%) |  | Often | 132 (3.8%) |
| Hui | 34 (1.0%) |  | Every day | 32 (0.9%) |
| Other ethnic minorities | 35 (1.0%) |  | **Drinking** |  |
| **Marital status** |  |  | Never | 2377 (69.0%) |
| Unmarried | 1483 (43.1%) |  | Occasionally | 915 (26.6%) |
| Married | 1780 (51.7%) |  | Often | 131 (3.8%) |
| Widowed | 66 (1.9%) |  | Every day | 20 (0.6%) |
| Divorced | 82 (2.4%) |  | **Monthly household income per capita** | |
| Separated | 32 (0.9%) |  | ≥¥5000 | 1885 (54.7%) |
| **Trait** |  |  | ¥3000-¥5000 | 1162 (33.7%) |
| Introverted | 890 (25.8%) |  | ¥1000-¥3000 | 330 (9.6%) |
| Extroverted | 1232 (35.8%) |  | <¥1000 | 66 (1.9%) |
| Mixed | 1321 (38.4%) |  | **Work pressure** |  |
| **Physical diseases** |  |  | Moderate | 2063 (59.9%) |
| No | 3135 (91.1%) |  | Intense | 1123 (32.6%) |
| Yes | 308 (8.9%) |  | Light | 257 (7.5%) |
| **Gynecological diseases** |  |  | **Major events in the last year** | |
| No | 3008 (87.4%) |  | No | 3215 (93.4%) |
| Yes | 435 (12.6%) |  | Yes | 228 (6.6%) |

**Supplemental Table 1** Continued

| (Continued) |  |  | (Continued) |  |
| --- | --- | --- | --- | --- |
| Variables | Total (N=3443) |  | Variables | Total (N=3443) |
| **Growth environment** | |  | **Living with parents** |  |
| Tier 1 cities | 743 (21.6%) |  | No | 1503 (43.7%) |
| Tier 2 cities | 1230 (35.7%) |  | Yes | 1940 (56.3%) |
| Tier 3 and below cities | 995 (28.9%) |  | **Living with chidren** |  |
| Rural areas | 475 (13.8%) |  | No | 2142 (62.2%) |
| **Place of residency** |  |  | Yes | 1301 (37.8%) |
| Tier 1 city | 745 (21.6%) |  | **Living with other relatives** |  |
| New tier 1 city | 788 (22.9%) |  | No | 3059 (88.8%) |
| Tier 2 city | 723 (21.0%) |  | Yes | 384 (11.2%) |
| Tier 3 city | 543 (15.8%) |  | **Living with friends** |  |
| Tier 4 city | 373 (10.8%) |  | No | 3098 (90.0%) |
| Tier 5 city | 191 (5.5%) |  | Yes | 345 (10.0%) |
| Other cities | 80 (2.3%) |  | **Living alone** |  |
| Overseas | 0 (0%) |  | No | 3188 (92.6%) |
| **Family structure** |  |  | Yes | 255 (7.4%) |
| Nuclear family | 2848 (82.7%) |  | **Living with other people** |  |
| Single-parent family | 351 (10.2%) |  | No | 3399 (98.7%) |
| Reorganized family | 188 (5.5%) |  | Yes | 44 (1.3%) |
| Broken family | 39 (1.1%) |  | **Number of children** |  |
| Others | 17 (0.5%) |  | 0 | 1678 (48.7%) |
| **Family relationship** |  |  | 1 | 1122 (32.6%) |
| Harmonious | 2670 (77.5%) |  | 2 | 524 (15.2%) |
| Average | 682 (19.8%) |  | >2 | 119 (3.5%) |
| Not harmonious | 91 (2.6%) |  | **Duration of stable relationship** |  |
| **Family burden** |  |  | ≥ 12 months | 2102 (61.1%) |
| Light | 1247 (36.2%) |  | 6-12 months | 693 (20.1%) |
| Medium | 1797 (52.2%) |  | 1-6 months | 648 (18.8%) |
| Heavy | 399 (11.6%) |  | **Dominant hand** |  |
| **Living with spouse** |  |  | Right hand | 2863 (83.2%) |
| No | 1774 (51.5%) |  | Left hand | 580 (16.8%) |
| Yes | 1669 (48.5%) |  |  |  |

**Supplemental Table 2** Distribution of options for self-reported factors of decreased sexual desire in participants (imputed)

| **Items** | Total (N=3443) |
| --- | --- |
| 1. **An operation, depression, injuries, or other medical condition** |  |
| No | 2297 (66.7%) |
| Yes | 1146 (33.3%) |
| 1. **Medication, drugs or alcohol you are currently taking** |  |
| No | 2536 (73.7%) |
| Yes | 907 (26.3%) |
| 1. **Pregnancy, recent childbirth, menopausal symptoms** |  |
| No | 2410 (70.0%) |
| Yes | 1033 (30.0%) |
| 1. **Other sexual issues you may be having (pain, decreased arousal or orgasm)** |  |
| No | 2071 (60.2%) |
| Yes | 1372 (39.8%) |
| 1. **Your partner’s sexual problems** |  |
| No | 2366 (68.7%) |
| Yes | 1077 (31.3%) |
| 1. **Dissatisfaction with your relationship or partner** |  |
| No | 2499 (72.6%) |
| Yes | 944 (27.4%) |
| 1. **Stress or fatigue** |  |
| No | 1536 (44.6%) |
| Yes | 1907 (55.4%) |

**Supplemental Table 3-A** Adjusted ddds ratios (aOR) in logistic regression models for decreased sexual desire due to Factor A (an operation, depression, injuries, or other medical condition)

| **Variables** | **Levels** | **aOR** | **CI** | **P** |
| --- | --- | --- | --- | --- |
| Current occupation  (ref = Student) | Farmers | 1.46 | (0.88, 2.39) | .137 |
|  | Workers | 2.09 | (1.46, 2.98) | **<.001** |
|  | Office staff | 1.30 | (1.03, 1.66) | **.031** |
|  | Civil servants | 1.65 | (1.22, 2.22) | **.001** |
|  | Professionals | 1.62 | (1.24, 2.11) | **<.001** |
|  | Self-employed | 0.93 | (0.62, 1.38) | .731 |
|  | Retired from illness | 1.53 | (0.55, 4.20) | .406 |
|  | Retired | 1.91 | (0.75, 4.71) | .160 |
|  | Unemployed | 1.01 | (0.43, 2.16) | .974 |
|  | Others | 0.62 | (0.27, 1.31) | .236 |
| Trait (ref = Introverted) | Extroverted | 0.89 | (0.73, 1.07) | .212 |
|  | Mixed | 0.73 | (0.60, 0.88) | **.001** |
| Smoking (ref = Never) | Occasionally | 1.57 | (1.23, 2.01) | **<.001** |
|  | Often | 1.12 | (0.74, 1.68) | .588 |
|  | Every day | 1.40 | (0.66, 2.97) | .374 |
| Drinking (ref = Never) | Occasionally | 1.17 | (0.97, 1.41) | .091 |
|  | Often | 1.64 | (1.09, 2.49) | **.019** |
|  | Every day | 1.04 | (0.39, 2.70) | .939 |
| Work pressure  (ref = Moderate) | Intense | 1.30 | (1.11, 1.52) | **.001** |
|  | Light | 0.99 | (0.73, 1.33) | .947 |
| Living with parents (ref = No) | Yes | 1.20 | (1.03, 1.41) | **.024** |
| Living with children (ref = No) | Yes | 1.14 | (0.96, 1.34) | .126 |
| Living alone (ref = No) | Yes | 1.27 | (0.95, 1.70) | .099 |
| Living with friends (ref = No) | Yes | 1.27 | (0.99, 1.62) | .056 |
| Living with others (ref = No) | Yes | 2.73 | (1.45, 5.09) | **.002** |
| Physical diseases (ref = No) | Yes | 1.86 | (1.40, 2.46) | **<.001** |
| Gynecological Disease (ref = No) | Yes | 1.54 | (1.21, 1.96) | **<.001** |

**Supplemental Table 3-B** Adjusted odds ratios (aOR) in logistic regression models for decreased sexual desire due to Factor B (medication, drugs or alcohol you are currently taking)

| **Variables** | **Levels** | **aOR** | **CI** | **P** |
| --- | --- | --- | --- | --- |
| Marriage  (ref = Unmarried) | Married | 1.52 | (1.18, 1.97) | **.001** |
|  | Widowed | 1.01 | (0.55, 1.82) | .978 |
|  | Divorced | 1.28 | (0.73, 2.22) | .390 |
|  | Separated | 0.78 | (0.33, 1.78) | .561 |
| Education  (ref = Middle school) | Out of school | 0.96 | (0.40, 2.32) | .921 |
|  | Primary school | 0.89 | (0.46, 1.74) | .741 |
|  | High school | 0.58 | (0.40, 0.83) | **.003** |
|  | University | 0.60 | (0.43, 0.85) | **.004** |
|  | Postgraduate | 0.45 | (0.30, 0.69) | **<.001** |
| Current occupation  (ref = Student) | Farmers | 0.70 | (0.38, 1.26) | .245 |
|  | Workers | 1.02 | (0.66, 1.55) | .944 |
|  | Office staff | 0.96 | (0.73, 1.27) | .770 |
|  | Civil servants | 1.48 | (1.06, 2.07) | **.022** |
|  | Professionals | 1.59 | (1.18, 2.16) | **.002** |
|  | Self-employed | 1.07 | (0.70, 1.63) | .761 |
|  | Retired from illness | 1.07 | (0.37, 3.07) | .903 |
|  | Retired | 1.32 | (0.49, 3.55) | .575 |
|  | Unemployed | 0.84 | (0.32, 1.93) | .695 |
|  | Others | 0.43 | (0.12, 1.13) | .123 |
| Trait (ref = Introverted) | Extroverted | 0.84 | (0.69, 1.03) | .097 |
|  | Mixed | 0.73 | (0.59, 0.90) | **.003** |
| Smoking (ref = Never) | Occasionally | 1.82 | (1.42, 2.31) | **<.001** |
|  | Often | 1.55 | (1.02, 2.34) | **.038** |
|  | Every day | 1.36 | (0.61, 2.94) | .441 |
| Work pressure  (ref = Moderate) | Intense | 1.29 | (1.08, 1.55) | **.005** |
|  | Light | 0.95 | (0.68, 1.30) | .753 |
| Drinking (ref = Never) | Left hand | 1.19 | (0.96, 1.48) | .108 |
| Growth environment  (ref = Tier 1 city) | Tier 2 cities | 0.94 | (0.76, 1.17) | .573 |
|  | Tier 3 and below cities | 0.80 | (0.63, 1.01) | .063 |
|  | Rural areas | 0.63 | (0.46, 0.86) | **.004** |
| Place of residence  (ref = Tier 1 city) | New tier 1 city | 1.39 | (1.07, 1.81) | **.013** |
|  | Tier 2 city | 1.32 | (1.01, 1.73) | **.045** |
|  | Tier 3 city | 1.55 | (1.17, 2.05) | **.002** |
|  | Tier 4 city | 1.41 | (1.02, 1.93) | **.034** |
|  | Tier 5 city | 1.55 | (1.05, 2.27) | **.025** |
|  | Other cities | 1.06 | (0.59, 1.86) | .831 |

**Supplemental Table 3-B** Continued

| **Variables** | **Levels** | **aOR** | **CI** | **P** |
| --- | --- | --- | --- | --- |
| Family structure  (ref = Nuclear family) | Single-parent family | 1.59 | (1.21, 2.09) | **.001** |
|  | Reorganized family | 1.83 | (1.29, 2.59) | **.001** |
|  | Broken family | 1.73 | (0.82, 3.56) | .139 |
|  | Others | 0.32 | (0.02, 1.67) | .283 |
| Family burden (ref = Light) | Medium | 0.85 | (0.71, 1.01) | .065 |
|  | Heavy | 0.77 | (0.57, 1.03) | .076 |
| Living with a partner (ref = No) | Yes | 0.80 | (0.63, 1.01) | .061 |
| Living with parents (ref = No) | Yes | 1.36 | (1.15, 1.62) | **<.001** |
| Living with children (ref = No) | Yes | 0.86 | (0.70, 1.05) | .131 |
| Living with friends (ref = No) | Yes | 1.38 | (1.05, 1.80) | **.019** |
| Physical diseases (ref = No) | Yes | 1.36 | (1.03, 1.78) | **.027** |

**Supplemental Table 3-C** Adjusted odds ratios (aOR) in logistic regression models for decreased sexual desire due to Factor C (pregnancy, recent childbirth, menopausal symptoms)

| **Variables** | **Levels** | **aOR** | **CI** | **P** |
| --- | --- | --- | --- | --- |
| Ethnic group (ref = Han) | Manchu | 0.56 | (0.34, 0.90) | **.018** |
|  | Hui | 0.49 | (0.19, 1.13) | .116 |
|  | Other ethnic orities | 1.08 | (0.48, 2.25) | .852 |
| Marriage  (ref = Unmarried) | Married | 1.53 | (1.22, 1.92) | **<.001** |
|  | Widowed | 2.03 | (1.15, 3.59) | **.015** |
|  | Divorced | 2.82 | (1.68, 4.74) | **<.001** |
|  | Separated | 1.43 | (0.64, 3.05) | .367 |
| Smoking (ref = Never) | Occasionally | 1.33 | (1.05, 1.68) | **.017** |
|  | Often | 1.22 | (0.81, 1.82) | .338 |
|  | Every day | 0.48 | (0.19, 1.10) | .101 |
| Dominant hand (ref = Right hand) | Left hand | 1.29 | (1.05, 1.58) | **.014** |
| Place of residence  (ref = Tier 1 city) | New tier 1 city | 1.15 | (0.92, 1.45) | .225 |
|  | Tier 2 city | 1.07 | (0.84, 1.35) | .597 |
|  | Tier 3 city | 1.03 | (0.80, 1.33) | .810 |
|  | Tier 4 city | 1.27 | (0.96, 1.68) | .098 |
|  | Tier 5 city | 0.83 | (0.57, 1.20) | .335 |
|  | Other cities | 0.54 | (0.29, 0.95) | **.038** |
| Living with parents (ref = No) | Yes | 1.22 | (1.04, 1.43) | **.017** |
| Living with children (ref = No) | Yes | 1.16 | (0.95, 1.43) | .152 |
| Living with relatives (ref = No) | Yes | 1.47 | (1.16, 1.87) | **.001** |
| Living with friends (ref = No) | Yes | 1.41 | (1.08, 1.82) | **.010** |
| Living with others (ref = No) | Yes | 1.71 | (0.83, 3.32) | .127 |
| Number of children (ref = 0) | 1 | 1.51 | (1.17, 1.95) | **.002** |
|  | 2 | 1.68 | (1.27, 2.20) | **<.001** |
|  | >2 | 1.90 | (1.22, 2.98) | **.005** |
| Major events in the past year (ref = No) | Yes | 1.32 | (0.97, 1.78) | .071 |
| Physical diseases (ref = No) | Yes | 1.54 | (1.19, 1.99) | **.001** |

**Supplemental Table 3-D** Adjusted odds ratios (aOR) in logistic regression models for decreased sexual desire due to Factor D (other sexual issues you may be having (pain, decreased arousal or orgasm))

| **Variables** | **Levels** | **aOR** | **CI** | **P** |
| --- | --- | --- | --- | --- |
| Current occupation  (ref = Student) | Farmers | 1.16 | (0.70, 1.88) | .560 |
|  | Workers | 1.33 | (0.93, 1.89) | .117 |
|  | Office staff | 1.28 | (1.02, 1.60) | **.034** |
|  | Civil servants | 1.47 | (1.10, 1.95) | **.008** |
|  | Professionals | 1.45 | (1.13, 1.88) | **.004** |
|  | Self-employed | 1.50 | (1.04, 2.15) | **.029** |
|  | Retired from illness | 0.42 | (0.13, 1.18) | .118 |
|  | Retired | 2.22 | (0.89, 5.59) | .084 |
|  | Unemployed | 0.50 | (0.19, 1.11) | .109 |
|  | Others | 0.61 | (0.29, 1.22) | .173 |
| Smoking (ref = Never) | Occasionally | 1.44 | (1.15, 1.80) | **.001** |
|  | Often | 1.39 | (0.94, 2.07) | .102 |
|  | Every day | 1.45 | (0.69, 3.02) | .320 |
| Monthly household income per capita  (ref = ≥¥5000) | ¥3000-¥5000 | 1.18 | (1.01, 1.39) | **.038** |
|  | ¥1000-¥3000 | 1.29 | (0.99, 1.67) | .057 |
|  | <¥1000 | 0.81 | (0.46, 1.38) | .446 |
| Work pressure  (ref = Moderate) | Intense | 1.40 | (1.20, 1.63) | **<.001** |
|  | Light | 0.99 | (0.74, 1.31) | .930 |
| Place of residence  (ref = Tier 1 city) | New tier 1 city | 1.38 | (1.11, 1.72) | **.003** |
|  | Tier 2 city | 1.11 | (0.88, 1.39) | .375 |
|  | Tier 3 city | 1.37 | (1.08, 1.74) | **.011** |
|  | Tier 4 city | 1.34 | (1.02, 1.75) | **.035** |
|  | Tier 5 city | 1.24 | (0.88, 1.74) | .222 |
|  | Other cities | 1.08 | (0.66, 1.76) | .749 |
| Family relationships  (ref = Harmonious) | Average | 1.21 | (1.00, 1.45) | **.046** |
|  | Not harmonious | 1.16 | (0.74, 1.81) | .513 |
| Living with children (ref = No) | Yes | 1.28 | (1.05, 1.57) | **.014** |
| Living with friends (ref = No) | Yes | 1.22 | (0.96, 1.55) | .099 |
| Number of children (ref = 0) | 1 | 0.89 | (0.71, 1.11) | .298 |
|  | 2 | 0.94 | (0.74, 1.20) | .646 |
|  | >2 | 0.57 | (0.36, 0.88) | **.012** |
| Major events in the past year (ref = No) | Yes | 1.54 | (1.16, 2.05) | **.003** |
| Gynecological Disease (ref = No) | Yes | 2.30 | (1.86, 2.85) | **<.001** |

**Supplemental Table 3-E** Adjusted odds ratios (aOR) in logistic regression models for decreased sexual desire due to Factor E (your partner’s sexual problems)

| **Variables** | **Levels** | **aOR** | **CI** | **P** |
| --- | --- | --- | --- | --- |
| Trait (ref = Introverted) | Extroverted | 0.86 | (0.71, 1.04) | .118 |
|  | Mixed | 0.74 | (0.61, 0.90) | **.002** |
| Drinking (ref = Never) | Occasionally | 1.18 | (0.99, 1.40) | .064 |
|  | Often | 1.55 | (1.05, 2.28) | **.026** |
|  | Every day | 1.01 | (0.38, 2.52) | .988 |
| Work pressure  (ref = Moderate) | Intense | 1.36 | (1.15, 1.59) | **<.001** |
|  | Light | 0.96 | (0.71, 1.29) | .791 |
| Drinking (ref = Never) | Left hand | 1.16 | (0.95, 1.42) | .149 |
| Place of residence  (ref = Tier 1 city) | New tier 1 city | 1.21 | (0.96, 1.52) | .105 |
|  | Tier 2 city | 1.00 | (0.79, 1.27) | .969 |
|  | Tier 3 city | 1.64 | (1.28, 2.10) | **<.001** |
|  | Tier 4 city | 1.39 | (1.05, 1.83) | **.020** |
|  | Tier 5 city | 1.63 | (1.15, 2.30) | **.006** |
|  | Other cities | 0.99 | (0.58, 1.66) | .983 |
| Family structure  (ref = Nuclear family) | Single-parent family | 1.35 | (1.05, 1.74) | **.021** |
|  | Reorganized family | 1.57 | (1.13, 2.17) | **.007** |
|  | Broken family | 1.74 | (0.88, 3.41) | .106 |
|  | Others | 1.72 | (0.61, 4.61) | .285 |
| Family relationships  (ref = Harmonious) | Average | 1.31 | (1.08, 1.58) | **.007** |
|  | Not harmonious | 1.11 | (0.69, 1.74) | .668 |
| Living with parents (ref = No) | Yes | 1.19 | (1.02, 1.39) | **.026** |
| Living with children (ref = No) | Yes | 1.22 | (1.05, 1.43) | **.011** |
| Living with friends (ref = No) | Yes | 1.21 | (0.95, 1.55) | .125 |
| Gynecological Disease (ref = No) | Yes | 1.70 | (1.37, 2.10) | **<.001** |

**Supplemental Table 3-F** Adjusted odds ratios (aOR) in logistic regression models for decreased sexual desire due to Factor F (dissatisfaction with your relationship or partner)

| **Variables** | **Levels** | **aOR** | **CI** | **P** |
| --- | --- | --- | --- | --- |
| Current occupation  (ref = Student) | Farmers | 1.26 | (0.73, 2.13) | .399 |
|  | Workers | 1.43 | (0.97, 2.09) | .065 |
|  | Office staff | 1.36 | (1.06, 1.76) | **.016** |
|  | Civil servants | 1.89 | (1.38, 2.58) | **<.001** |
|  | Professionals | 1.97 | (1.50, 2.60) | **<.001** |
|  | Self-employed | 1.59 | (1.06, 2.36) | **.022** |
|  | Retired from illness | 1.06 | (0.37, 2.89) | .904 |
|  | Retired | 2.99 | (1.17, 7.69) | **.021** |
|  | Unemployed | 0.76 | (0.27, 1.79) | .555 |
|  | Others | 1.24 | (0.55, 2.56) | .585 |
| Trait (ref = Introverted) | Extroverted | 0.89 | (0.73, 1.08) | .233 |
|  | Mixed | 0.71 | (0.58, 0.86) | **.001** |
| Smoking (ref = Never) | Occasionally | 1.63 | (1.29, 2.07) | **<.001** |
|  | Often | 1.68 | (1.13, 2.49) | **.010** |
|  | Every day | 3.33 | (1.59, 7.17) | **.002** |
| Work pressure  (ref = Moderate) | Intense | 1.37 | (1.15, 1.62) | **<.001** |
|  | Light | 1.30 | (0.95, 1.76) | .092 |
| Drinking (ref = Never) | Left hand | 1.22 | (0.99, 1.51) | .064 |
| Family structure  (ref = Nuclear family) | Single-parent family | 1.41 | (1.08, 1.84) | **.012** |
|  | Reorganized family | 1.61 | (1.15, 2.26) | **.006** |
|  | Broken family | 1.08 | (0.52, 2.18) | .831 |
|  | Others | 0.61 | (0.14, 1.97) | .457 |
| Family relationships  (ref = Harmonious) | Average | 1.29 | (1.05, 1.58) | **.014** |
|  | Not harmonious | 1.29 | (0.80, 2.05) | .292 |
| Living with parents (ref = No) | Yes | 1.59 | (1.34, 1.89) | **<.001** |
| Living with friends (ref = No) | Yes | 1.50 | (1.16, 1.93) | **.002** |
| Living alone (ref = No) | Yes | 1.35 | (1.00, 1.81) | .051 |
| Living with others (ref = No) | Yes | 1.84 | (0.9, 3.58) | .080 |
| Major events in the past year (ref = No) | Yes | 1.46 | (1.08, 1.97) | **.014** |
| Gynecological Disease (ref = No) | Yes | 1.60 | (1.27, 2.00) | **<.001** |

**Supplemental Table 3-G** Adjusted odds ratios (aOR) in logistic regression models for decreased sexual desire due to Factor G (stress or fatigue)

| **Variables** | **Levels** | **aOR** | **CI** | **P** |
| --- | --- | --- | --- | --- |
| Marriage  (ref = Unmarried) | Married | 1.33 | (1.12, 1.58) | **.001** |
|  | Widowed | 0.92 | (0.51, 1.62) | .769 |
|  | Divorced | 1.34 | (0.79, 2.29) | .280 |
|  | Separated | 2.20 | (0.99, 5.13) | .059 |
| Education  (ref = Middle school) | Out of school | 2.08 | (0.85, 5.51) | .119 |
|  | Primary school | 1.49 | (0.77, 2.94) | .239 |
|  | High school | 1.11 | (0.79, 1.56) | .556 |
|  | University | 1.52 | (1.10, 2.11) | **.011** |
|  | Postgraduate | 1.86 | (1.28, 2.73) | **.001** |
| Current occupation  (ref = Student) | Farmers | 0.75 | (0.45, 1.26) | .285 |
|  | Workers | 1.05 | (0.72, 1.53) | .796 |
|  | Office staff | 1.36 | (1.07, 1.71) | **.011** |
|  | Civil servants | 0.87 | (0.65, 1.17) | .346 |
|  | Professionals | 1.18 | (0.91, 1.53) | .210 |
|  | Self-employed | 0.72 | (0.50, 1.05) | .091 |
|  | Retired from illness | 0.69 | (0.24, 1.92) | .479 |
|  | Retired | 1.61 | (0.63, 4.19) | .319 |
|  | Unemployed | 0.51 | (0.23, 1.04) | .070 |
|  | Others | 0.92 | (0.47, 1.89) | .824 |
| Trait (ref = Introverted) | Extroverted | 0.87 | (0.72, 1.05) | .148 |
|  | Mixed | 1.13 | (0.94, 1.37) | .194 |
| Smoking (ref = Never) | Occasionally | 0.85 | (0.66, 1.10) | .219 |
|  | Often | 0.49 | (0.32, 0.74) | **.001** |
|  | Every day | 1.37 | (0.62, 3.21) | .453 |
| Drinking (ref = Never) | Occasionally | 1.55 | (1.29, 1.87) | **<.001** |
|  | Often | 1.62 | (1.05, 2.50) | **.029** |
|  | Every day | 0.77 | (0.28, 2.06) | .603 |
| Work pressure  (ref = Moderate) | Intense | 1.86 | (1.59, 2.19) | **<.001** |
|  | Light | 0.77 | (0.58, 1.02) | .070 |
| Growth environment  (ref = Tier 1 city | Tier 2 cities | 1.15 | (0.94, 1.40) | .163 |
|  | Tier 3 and below cities | 1.34 | (1.08, 1.65) | **.007** |
|  | Rural areas | 1.35 | (1.04, 1.75) | **.023** |
| Place of residence  (ref = Tier 1 city) | New tier 1 city | 0.94 | (0.75, 1.18) | .595 |
|  | Tier 2 city | 0.67 | (0.53, 0.85) | **.001** |
|  | Tier 3 city | 0.79 | (0.62, 1.02) | .068 |
|  | Tier 4 city | 1.15 | (0.87, 1.52) | .318 |
|  | Tier 5 city | 0.77 | (0.54, 1.08) | .131 |
|  | Other cities | 0.61 | (0.37, 1.00) | .052 |

**Supplemental Table 3-G** Continued

| **Variables** | **Levels** | **aOR** | **CI** | **P** |
| --- | --- | --- | --- | --- |
| Family relationships  (ref = Harmonious) | Average | 1.21 | (1.00, 1.47) | .053 |
|  | Not harmonious | 1.37 | (0.85, 2.22) | .199 |
| Living with relatives (ref = No) | Yes | 0.84 | (0.67, 1.06) | .139 |
| Major events in the past year (ref = No) | Yes | 1.65 | (1.20, 2.28) | **.002** |
| Gynecological Disease (ref = No) | Yes | 1.78 | (1.42, 2.26) | **<.001** |
